# Rapid expulsion of microswimmers by a vortical flow

**DOI:** 10.1038/ncomms11114

**Published:** 2016-03-23

**Authors:** Andrey Sokolov, Igor S. Aranson

**Affiliations:** 1 Materials Science Division, Argonne National Laboratory, Argonne, Illinois 60439, USA; 2 Department of Engineering Sciences and Applied Mathematics, Northwestern University, 2145 Sheridan Road, Evanston, Illinois 60202, USA

## Abstract

Interactions of microswimmers with their fluid environment are exceptionally complex. Macroscopic shear flow alters swimming trajectories in a highly nontrivial way and results in dramatic reduction of viscosity and heterogeneous bacterial distributions. Here we report on experimental and theoretical studies of rapid expulsion of microswimmers, such as motile bacteria, by a vortical flow created by a rotating microparticle. We observe a formation of a macroscopic depletion area in a high-shear region, in the vicinity of a microparticle. The rapid migration of bacteria from the shear-rich area is caused by a vortical structure of the flow rather than intrinsic random fluctuations of bacteria orientations, in stark contrast to planar shear flow. Our mathematical model reveals that expulsion is a combined effect of motility and alignment by a vortical flow. Our findings offer a novel approach for manipulation of motile microorganisms and shed light on bacteria–flow interactions.

## Abstract (web summary)

The control of microswimmers such as bacteria is important for emerging applications of active bioinspired materials. Here, the authors demonstrate the use of vortical shear to expel suspended motile bacteria from the vicinity of a rotating microparticle.

## Graphical abstract

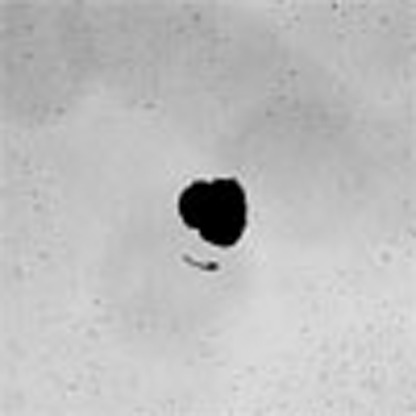

Interactions of bacteria with fluid and solid surfaces are exceptionally complex and are the topic of active research^1^,^2^,^3^,^4^,^5^,^6^,^7^,^8^,^9^. In addition to a variety of chemical and physical factors, swimming trajectories are also affected by macroscopic shear flow. Migration of microorganisms in fluid or over surfaces is determined by a multitude of various physical and biological processes, such as chemotaxis^10^,^11^, thermotaxis^12^, quorum sensing^13^, gyrotaxis^14^,^15^ and rheotaxis^6^,^16^. Many bacteria, including *Escherichia coli* and *Bacillus subtilis*, undergo a biased random walk consisting of smooth swimming segments and tumbles (abrupt changes of the swimming direction)^17^. That behaviour enables them to move up to a chemoattractant or in the direction of the temperature gradient. A drift of bacteria can also be a result of a combination of different mechanisms, such as chemotaxis and rheotaxis^11^. In contrast to chemotaxis and thermotaxis, which are fairly well characterized both biochemically and mathematically^10^,^18^, gyrotaxis^14^,^15^ and rheotaxis^5^,^6^,^16^ of microorganisms are purely physical phenomena: no chemical/temperature stimuli or active receptor response are engaged. In the course of gyrotaxis, a preferred orientation of a bottom-heavy cell is determined by a balance between the viscous torque exerted by a fluid and the gravitational torque caused by the asymmetric mass distribution. Bacterial rheotaxis occurs due to a combination of two effects: reorientation of elongated bacterial bodies by shear flow and self-propulsion due to flagella rotation. This mechanism does not require any active sensing in contrast with fish rheotaxis^19^. Microfluidic experimental and theoretical studies of sheared suspensions of swimming bacteria^5^,^6^,^20^ demonstrated that a planar shear flow triggers a bacterial drift and alters their spatial distribution function. Particularly, the distribution function of bacteria in a microfluidic channel has a minimum along the channel center line corresponding to zero local shear. The observed microswimmer transport in ref. 5 also required significant intrinsic stochasticity of bacterial orientations characterized by the rotational diffusion coefficient *D*_r_. Accordingly, the spatial distribution function remains homogeneous if the rotational diffusion *D*_r_ is set to zero. Stochasticity of bacterial orientations is also needed for the explanation of viscosity reduction in suspensions of microswimmers in a planar shear flow^21^,^22^. This result is somewhat counterintuitive, because a non-tumbling strain of *B. subtilis* was used in ref. 23. Scattering and trapping of deformable microswimmers in a swirling flow was investigated numerically in ref. 24. Numerical studies of bacteria dynamics in non-planar flow represented by a periodic array of vortices demonstrated an accumulation of swimmers near separatrices dividing the vortices^25^.

The local flow environment of motile bacteria is not generally represented by a planar shear flow. A typical turbulent flow is manifested by recurring vortices and non-zero curvature of the flow lines. Moreover, in experiments^23^,^26^, the shear flow created by a rotating particle, is characterized by a vortical motion and closed streamlines.

Here we show that the vortical structure of the flow, and, correspondingly, curvature of the streamlines has a highly nontrivial effect not present in a planar shear flow. We find that the spatial distribution of bacteria in a vortical flow created by a rotating particle is strongly heterogeneous with an almost complete concentration depletion in the vicinity of the rotational center *r*=0, that is, a shear-rich area. The formation of macroscopic depletion region occurs surprisingly fast, with the time scale determined by the bacterial swimming speed. Our mathematical model reveals that, in stark contrast to a planar shear, no rotational noise is needed for the onset of depletion. We calculate the distribution functions for position and orientation of bacteria. Our theoretical findings match well with the experimental data.

## Results

### Formation of a depletion zone

**Figure 1:**
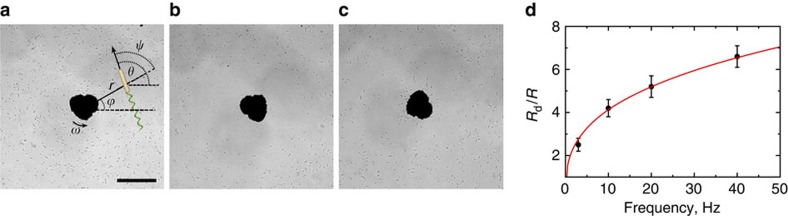
Formation of a depletion zone. Distribution of bacteria in the vicinity of the rotating particle. Bacteria imaged approximately 0–5 μm above the surface of the sessile drop. Scale bar, 100 μm. Rotation frequency is 20 Hz. (**a**) Illustration of bacterial orientation one second after the start of rotation and the chosen coordinate system. (**b**) A stationary distribution of bacteria and a depletion zone: concentration of swimming bacteria around the rotating particle is reduced. (**c**) A depletion zone fades away after cessation of rotation. Bacteria trapped by the rotating particle are released. (**d**) Radius of the depletion zone *R*_d_ normalized by the particle radius *R*=30 μm versus frequency. The value of *R*_d_ is determined from the condition that at *r*=*R*_d_ the concentration of bacteria is one half of the equilibrium concentration. Error bars (s.d.) are calculated over a sequence of 10 non-consecutive frames. Solid red line is the fit to equation (7), *R*_d_∝*ω*^1/3^.

In our experiment, a pendent drop of bacterial suspension (10 μl) is attached to a microscope glass slide, see Methods. A nickel particle of radius *R*≈30 μm is placed in the center of the drop, and the gravity force keeps the particle around the nadir. Two pairs of orthogonal Helmholtz coils create a uniform rotating magnetic field forcing the particle to spin with the field frequency. If the magnetic field is turned off, then bacteria are swimming randomly in the vicinity of the particle and their spatial and angular distributions are homogeneous. When a rotating magnetic field with frequency 2–40 Hz is applied, the spinning particle creates a macroscopic vortex with a diameter of a few hundred microns. This vortex advects bacteria along streamlines and aligns their bodies parallel to the local flow direction (Fig. 1a). Rarely bacteria flip their orientations. While the spatial distribution (during first few seconds) is almost uniform, the orientational distribution acquires two sharp peaks near relative angle 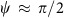 and 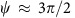, see Fig. 1a for the definition of relative angle 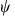. Within a few seconds the concentration of motile bacteria in the vicinity of the particle starts to decrease resulting in a formation of a macroscopic depletion zone. The depletion zone expands over a few minutes (Fig. 1b). The trajectories of bacteria are represented by unwinding spirals with a slowly changing curvature. Since the shear flow created by a spherical particle is decaying roughly as 1/*r*^2^ with the distance *r* from the origin, see Supplementary Fig. 1, at a certain distance the rotational diffusion of bacteria overcomes the shear-induced alignment. That limits the depletion zone diameter. For not too high concentrations, most of the bacteria are confined to a thin layer near the surface of the drop due to hydrodynamic attraction/steric collision^27^,^28^,^29^. This confinement allows an effective two-dimensional approximation for the interaction between bacteria and flow created by the rotating particle.

### Trapping of bacteria

Some bacteria become trapped by the flow around the rotating particle. Due to the visual overlapping of these bacteria with the rotating particle we were not able to track their orientations and positions. Their presence is revealed after the cessation of rotation. Once the macroscopic flow terminates, trapped bacteria swim away from the particle (Fig. 1c). In the absence of rotation, the depletion zone fades away, see Supplementary Movies 1 and 2. The number of trapped bacteria *N*_trap_ increases with the rotation frequency and is roughly proportional to the depletion area, see Table 1. We observed that ∼40% of the bacteria initially located in the depletion zone become trapped. Width of the depletion zone *R*_d_ increases with the rotation frequency, see Fig. 1d.

### Effect of collective behaviour on the depletion

**Figure 2:**
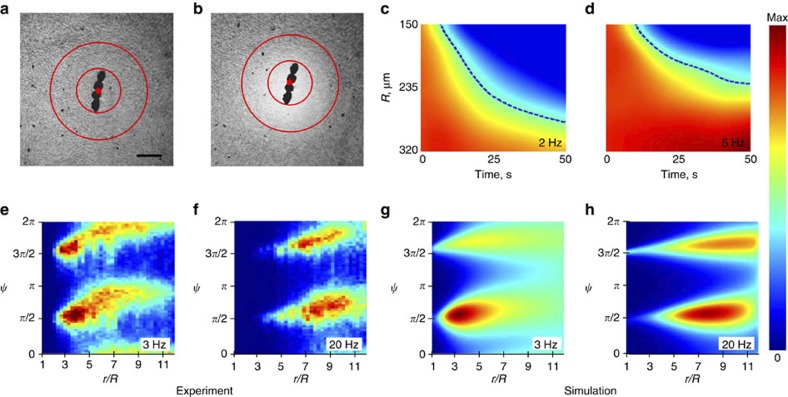
Space time diagrams and probability distribution functions for bacterial orientations. (**a**–**d**) A formation of a depletion zone at high bacterial concentration. (**a**) Initial distribution of bacteria. (**b**) Distribution of bacteria 50 s after the onset of rotation. White colour corresponds to a lower concentration of bacteria. Rotation frequency is 5 Hz. (**c**,**d**) Space-time diagram for bacteria distribution between two red circles in **a**,**b**. Results are presented for two rotation frequencies: 2 Hz (**c**) and 5 Hz (**d**). Dashed lines show edges of depletion areas as a function of time. Vertical scales on plots (**c**,**d**) are the same. (**e**–**h**) Stationary bacterial distribution functions 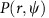. Experimental (**e**,**f**) and simulation (**g**,**h**) results are presented for two frequencies of rotation: 3 and 20 Hz. Rotational diffusion is assumed *D*_r_=0.1 rad^2^ s^−1^ and average swimming speed *V*_0_=15 μm s^−1^.

Experimental results for higher bacterial concentrations *n*≈10^10^ cm^−3^ are shown in Fig. 2. At such concentrations bacteria swim collectively, which is manifested by the formation of large scale rapidly moving (up to 60 μm s^−1^) structures with the typical scale of *L*=30–40 μm (refs 3, 4, 7). The scale of these structures is comparable with the size of the rotating particle and the characteristic flow scale. To examine the interaction of collectively swimming bacteria with the circular flow, a bigger vortex was created. We placed four permanently magnetized nickel particles into the bacterial suspension. Due to ferromagnetic interaction, the particles formed a chain (Fig. 2a,b). Its geometrical shape remained constant in the course of rotation. The formation of a depletion zone around the rotating chain was observed at a wide range of rotation rates *f*=2–50 Hz, see Supplementary Movies 3 and 4. We display a space–time diagram by stacking average bacterial concentration distributions at a distance *r* from the origin at different moments of time *t* (measured from the onset of circular flow). Since the flow in the vicinity of the rotating chain is not truly two-dimensional, we discarded the region of *r*<150 μm. From the space–time diagram one extracts the expansion rate of the depletion zone, Fig. 2c,d. Strikingly, bacteria migrate from the center of rotation faster in the case of a slower vortex. This observation highlights that the expulsion of bacteria is not caused by the centrifugal forces but rather a non-trivial interplay between shear induced alignment and self-propulsion. This experiment also affirms that formation of the depletion zone is due to an interaction between individual bacteria and shear flow rather than a collective effect. Collective behaviour of bacteria results in a dramatic increase of effective fluctuations^30^ which, in turn, decreases the depletion magnitude created by a single rotating particle, see Supplementary Movie 5.

### Non-motile bacteria

We performed the control experiments with non-motile (dead) bacteria. The bacteria were killed by a small amount of alcohol mixed with the bacterial suspension. According to our observations, bacteria exposed to alcohol do not swim while preserving their elongated body shape after the alcohol evaporation. The orientational distribution of dead bacteria in a vortex looks very similar to the distribution of their motile counterparts. However, the spatial distribution remains uniform (see Supplementary Movie 6). This confirms that formation of the depletion zone is a result of coupling between the shear flow and the bacterial motility.

## Discussion

We characterize the configuration of bacteria by a single-particle distribution function 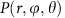 of the bacterial position (*r*, 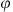) and the bacterial orientation *θ*. Here *r*, 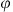 are polar coordinates with the origin at the center of rotation. Interactions between bacteria do not alter the underlying mechanism of a depletion zone formation: we observed similar effects with a wide range of bacterial concentrations. At low concentrations of bacteria, bacterial bodies are not overlapping, and each bacterium in the microscope field of view can be tracked. Using a custom script (based on MATLAB toolboxes), we measured positions, orientations and velocities of bacteria at every moment in time. Detection of bacterial orientation 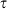 poses a uncertainty ±*π* due to visual degeneracy between a tail and a head of a bacterium. To overcome this limitation we developed a dedicated tracking algorithm, see Methods.

**Figure 3:**
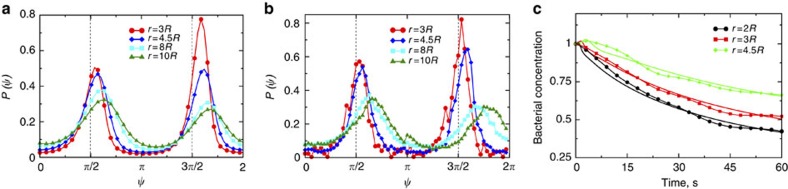
Comparison of results for orientation distributions and concentration evolution. Orientational distributions of bacteria at various distances *r* from the center of rotation obtained from simulations (**a**) and experiments (**b**). Rotation frequency of the particle is 20 Hz. (**c**) Relaxation of bacterial concentration at different distances from the rotating particle. Lines with symbols represent the experimental data and lines without symbols are simulation results for *V*_0_=25 μm s^−1^, *R*=30 μm, *D*_r_=0.1 s^−1^. Rotation frequency is 10 Hz.

The results are summarized in Figs 2e,f and 3. The orientational distribution function *P*(*r*, 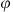) has two distinct peaks in the vicinity of *π*/2 and 3*π*/2, 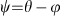 is a relative angle (Fig. 1a). Due to the bacterial motility, the distribution is not symmetrical and locations of the peaks shift towards bigger values. This effect has a simple explanation. For non-motile elongated particles and in the absence of noise, the shear-induced reorientation results in two peaks at 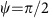 and 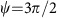, that is, they tend to align parallel to streamlines. In a planar shear flow and in the absence of rotational noise, motile bacteria maintain their average positions with respect to flow transverse direction^31^. However, due to curvature of the streamlines in a vortex flow, motile bacteria swim into regions with different flow orientation. Thus, self-propulsion results in misalignment and a systematic migration across circular streamlines. Such a drift mechanism does not require a non-zero rotational diffusion of bacteria crucial in ref. 5. Moreover, since the migration of bacteria is controlled by the rotation rate of the particle and the bacterial swimming speed, the expulsion effect is observed in the absence of diffusion (or angular noise). The characteristic drift speed and the fraction of bacteria expelled from the center of rotation (practically complete depletion) can significantly exceed the corresponding values observed in a planar flow (about 70% in the middle of the channel^5^).

To quantify the observed effects, we developed a kinetic model of swimming bacteria in a vortical flow of viscous incompressible fluid created by a solid rotating particle of radius *R*. Far from the particle, 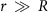, the flow is close to the two-dimensional, 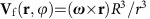, where ***ω***=2*πf***z**_0_ is particle's angular velocity, ***z***_0_ is a vertical unit vector. We model bacteria as self-propelled rigid prolate ellipsoids immersed in the external flow **V**_f_(*r*, 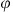). In the zero Reynolds number limit (Stokes flow) the orientation of a single bacterium obeys Jeffery's equation^32^,^33^


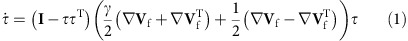


For an elongated particle, such as a bacterium, the shape parameter *γ*≈1 (we set *γ*=1 for simplicity). The bacterial orientation 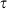 can be characterized by the angle *θ*, see Fig. 1a. Due to self-propulsion, the total speed of a bacterium *v* may be written as 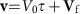, where *V*_0_ is the bacterial swimming speed. Then bacterium's equations of motion in the flow **V**_f_ (*r*, 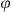) are of the form


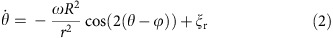



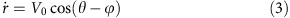



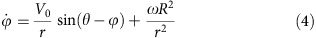


where *ξ*_r_ is an uncorrelated noise with the intensity 〈*ξ*_r_(*t*)*ξ*_r_(*t*′)〉=2*D*_r_*δ*(*t*−*t*′) accounting for random orientation fluctuations. Correspondingly, an equation for a relative angle 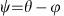 is of the form


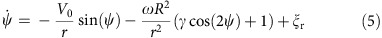


In the absence of fluctuations (*D*_r_=0), equation (5) has 2 fixed points 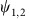, which for 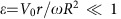 (shear dominates propulsion) are given by 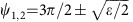. For example, for our experimental conditions, *R*=30 μm, *f*=20 Hz, *V*_0_∼20–30 μm s^−1^, we obtain *ɛ*≈0.008 at *r*=*R*. The fixed point 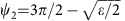 is unstable, and stable fixed point is of the form


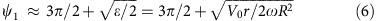


The stable point describes the bacteria swimming away from the origin with the radial velocity 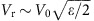. This effect explains the expulsion of bacteria by a vortical flow and the onset of outward flux of bacteria due to rotation of the particle. With the fluctuations present (*D*_r_≠0), the stable fixed point generates a sharp peak in the orientation distribution. Moreover, the position of the peak will move toward large angles with the increase of *r* (see Figs 2e–h and 3a,b), in agreement with our experimental data shown in Fig. 3b. The effect is not present for passive particles or non-motile bacteria (*V*_0_=0).

Width of the depletion zone *R*_d_ can be estimated from the analysis of linearized equation (5). Let us compare the relaxation rate 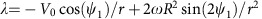 towards the stable equilibrium 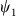 with the rotational diffusion *D*_r_. In the limit of 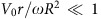 we obtain that 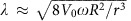. From the condition 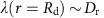 we find


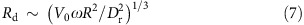


This expression qualitatively agrees with our experimental data, see Fig. 1d.

To understand the effect of fluctuations, we used the Fokker-Planck equation for the probability distribution 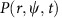 corresponding to equations (2, 3, 4), see Methods. equation (9) was integrated numerically. The results are presented in Figs 2g,h and 3. Excellent qualitative agreement with the experimental results was observed. As in the experiment, the distribution function has two broad peaks near 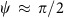 and 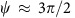. The peaks fade with the increase of distance *r* and the distribution function flattens.

Figure 3c displays the temporal evolution of bacterial concentration at different distances from the rotation particle. While bacterial concentration monotonically decreases over time for small radii *r*≲3*R*, it may temporarily increase for large radii *r*⪞4*R*, which is reflected by a small peak at *t*≈4–5 s in Fig. 3c. This temporal increase is caused by a migration of motile bacteria from smaller radii. The time scales of presented functions also depend on the distance from the rotation origin. Formation of the propagating concentration peaks was also seen in the solution of the Fokker–Planck equation (9), see Fig. 3c and Supplementary Fig. 2. Concentration decreases faster at small radii. The overall good agreement with theoretical result is observed. We attribute the differences between experimental data and simulation results to several factors, mostly to a three-dimensional structure of the flow created by the rotating spherical particle and a noise created by imperfections in the particle shape.

Our results elucidate the subtle role of fluctuations in the experiments with effective viscosity of active suspensions^23^,^26^. Namely, the observed sevenfold viscosity reduction cannot be easily explained by the intrinsic stochasticity in bacterial orientations for a planar shear flow, provided that non-tumbling strains of bacteria was used. This effect can be illustrated as follows. The effective viscosity *η*_eff_ is defined as a ratio of a shear stress component *σ*^sh^ to the corresponding rate of strain component 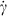. In the case of two-dimensional rotational flow with the azimuthal velocity 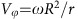, the rate of strain is 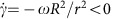, and the effective viscosity is of the form 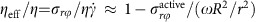. In the cylindrical coordinate system (see Fig. 1a), the corresponding component of the shear stress tensor 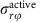 associated with the motility of bacteria (so-called active stress, normalized by solvent viscosity *η*) can be written as (see Supplementary Note 3)


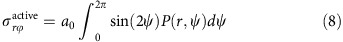


where *a*_0_ is proportional to the strength of the hydrodynamic dipole (stresslet) imposed by bacteria on the fluid and increases with the increase in swimming speed *V*_0_. For bacteria (that is, pushers) the sign of *a*_0_ is negative, *a*_0_<0 (refs 21, 22, 34). In the absence of bacteria orientation fluctuations (for example, tumbling), the steady-state probability distribution function 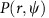 is reduced to a *δ*-function, 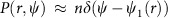, where 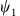 is the stable fixed point given by equation (6) and *n* is the local bacterial concentration. Thus, substituting this expression for 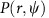 into equation (8), we readily obtain 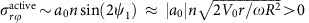. Since the active stress contribution is positive, while the strain rate is negative, we obtain the overall reduction of the effective viscosity. For very small strain rates, 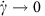, the effective viscosity *η*_eff_→−∞. However, this limit is singular, and an infinitesimally small rotational diffusion *D*_r_ will regularize the divergence^21^. Thus, in the case of a flow created by a rotating particle, such as was used in refs 23, 26, vortical structure of the flow results in strong asymmetry of the orientation distribution function, even in the absence of tumbling, and, in turn, a noticeable viscosity reduction. We estimated the effective viscosity reduction for the conditions of the experiment^23^, that is, concentration *n*≤10^10^ cm^−3^ (that is, slightly below the threshold for the onset of collective behaviour when interactions between the bacteria still can be neglected), and the rotation rate 0.5 Hz. An estimate for the dipolar moment strength can be found in ref. 29. We obtain that relative reduction of the effective viscosity due to this effect is of the order of 20–30%, see Supplementary Note 3. These values are below what was observed experimentally. Further reduction of the effective viscosity is likely due to additive effects of fluctuations and hydrodynamic interactions between the bacteria^21^,^35^.

Let us briefly discuss a connection between two seemingly different phenomena: reduction of the effective viscosity and depletion of bacterial concentration in active suspensions. While both phenomena occur in the same system, they are manifested at different experimental conditions. Reduction of the viscosity is observed at low shear rates (since for very high shear rates the passive contribution to the stress tensor dominates over active stress *σ*^active^), while the depletion occurs at much higher shear rates. Yet, the origin of both phenomena is similar and is associated with a non-isotropic orientation distribution of bacteria in a vortical flow. Formally, no rotation diffusion is needed for the onset of depletion and viscosity reduction. However, angular diffusion plays a significant role at low shear rates, making corresponding angular distribution functions relatively wide. For that reason, directed migration of bacteria and formation of a depletion zone was suppressed in experiments^23^,^26^.

In conclusion, we demonstrated, both experimentally and theoretically, that a rapidly rotating microparticle expels microswimmers in its vicinity. In contrast to planar shear flow^5^, the expulsion does not require stochasticity in microswimmer orientation and occurs on a much faster time scale. Our results shed new light on the bacterial dynamics in vortical flows, which are ubiquitous in bacterial habitats. Finally, elucidation of the physical mechanisms governing response of microswimmers on shear and the role of intrinsic fluctuations may lead to better understanding of active materials behaviour^9^,^36^. Our findings can be used to prevent biofilm formation/biofouling in microfluidic devices.

## Methods

### Bacteria preparation

Bacteria *B. subtilis* (strain 1085) were inoculated on a LB agar plate (Sigma-Aldrich) and stored in a refrigerator at temperature 4 °C. Twelve to 14 hours before the experiment bacteria were extracted from an isolated colony and transferred to a 15 ml plastic tube filled with Terrific Broth growth medium and placed in a shaking incubator. For an optimal growth rate bacteria were incubated at 35 °C. The concentration of bacteria during incubation period was monitored by measuring the optical opacity of the suspension with an infrared proximity sensor. At the end of their exponential stage, the bacteria were extracted from the growth medium by centrifugation and washed.

### Tracking algorithm

Our algorithm allows measuring the velocities of bacteria **V**_b_ and the magnitude of vortex flow **V**_f_ at any position and at any moment in time. The algorithm allows determining the velocity of a bacterium with respect to the fluid, **V**_b_−**V**_f_. We assumed that in a dilute suspension each bacterium swims into the direction of its orientation relative to the local flow. In contrast with ref. 37, our system is not confined by the walls. Thus, at low concentrations, the interactions between the bacterium and the flow generated by its neighbors are negligible. As in ref. 37, the bacterial orientations also appeared to be slightly larger than 3*π*/2 with respect to the radius vector. From two possible bacterial orientations 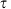 and 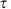
+*π*, we select the one that satisfies the condition 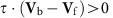. The flow 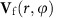 was estimated simultaneously by measuring velocities of vertically oriented bacteria. Such bacteria appear on camera images (horizontal projection of bacterial bodies) as particles with the aspect ratio *α* close to 1, while horizontally oriented bacteria has the aspect ratio *α*=7–10. For the practical implementation, we assumed that bacteria are vertically oriented if *α*<2. The horizontal component of their velocities with respect to the fluid is negligible; however, they are advected horizontally by the macroscopic flow created by the rotating particle. Tracking such bacteria allows reconstructing the flow field. Since the macroscopic flow is time-independent over the entire duration of our experiment (except the very first moment), we time averaged the velocities of the vertical oriented bacteria at each position 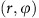. This approach allowed measuring 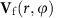 accurately enough to reconstruct the individual velocities of bacteria relative to the fluid.

### Fokker-Planck equation

The Fokker–Planck equation for the evolution of the probability distribution function 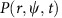 in polar coordinates is of the form


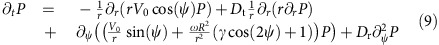


A translational diffusion *D*_t_ is introduced for the regularization of the steady-state solution. equation (9) was solved numerically in a rectangular [0; 2*π*] × [*R*; *L*] domain, 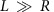, where *R* is the radius of the rotating particle. 2*π* periodicity condition was imposed in the 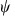 direction. A no-flux condition 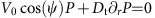 was set at *r*=*R* and *r*=*L*. Mesh points of dimension 400 × 200 were typically used. The swimming speed *V*_0_ was chosen in the range *V*_0_=15–25 μm s^−1^. The length was scaled by particle radius *R*=30 μm, and time by *t*_0_=*R*/*V*_0_≈1.2–2 s. We set rotational diffusion *D*_r_=0.1 rad^2^ s^−1^, which is consistent with our measurements for this strain of *B. subtilis*^7^. The translation diffusion arises from the fact that swimming speed of each bacterium fluctuates. From our previous study^38^ we estimate the standard deviation of the swimming speed Δ*V*≈4–5 μm s^−1^ and typical correlation time Δ*t*=3 s. The estimate yields the value of transnational diffusion *D*_t_≈Δ*V*^2^Δ*t*≈50–75 μm^2^ s^−1^. The effect of the translational diffusion on stationary concentration profiles is illustrated in Supplementary Fig. 3. Reduction of *D*_t_ leads to a deeper depletion of concentration near the particle. The corresponding values of dimensionless parameters in equation (9) are: 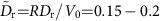, 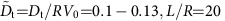. The growth of the depletion zone is limited by rotational diffusion of bacteria. At distances 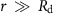, see equation (7), random fluctuations of bacterial orientations dominate over the shear alignment.

Stationary one-dimensional (1D) solution to equation (9) is obtained by neglecting the terms containing derivatives with respect to *r*, see Supplementary Note 1 and Supplementary Fig. 4. This approximation is valid if the dependence on *r* is slow compared to the dependence on 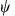, which is a case for 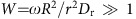. The shift of the distribution function peaks 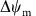 is described in this limit as following, see Supplementary Note 2:


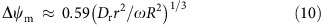


Equation (10) is indeed consistent with the experimental data in Fig. 3a,b for *r*/*R*≲3.5, see Supplementary Fig. 5. The shape of the probability distribution is described by function given in Supplementary Fig. 6. The fit yields the rotational diffusion *D*_r_≈0.1 rad^2^ s^−1^. For larger *r* values the 1D approximation becomes inaccurate, and the shift of the peaks 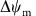 is roughly twice smaller than in the full equation for chosen parameters. Thus, using the 1D solution in this range of radii would yield anomalously large value of *D*_r_≈1 rad^2^ s^−1^.

## Additional information

**How to cite this article:** Sokolov, A. *et al*. Rapid expulsion of microswimmers by a vortical flow. *Nat. Commun.* 7:11114 doi: 10.1038/ncomms11114 (2016).

## Supplementary Material

Supplementary Information Supplementary Figures 1-6, Supplementary Notes 1-3 and Supplementary References

Supplementary Movie 1 Reorientation of bacteria by a vortex shear flow leads to rapid expulsion of bacteria from the rotating particle. The rotation frequency is 20 Hz. A small fraction of bacteria is trapped by the particle. These bacteria are released after the cessation of rotation. The movie is sped up by a factor of 10.

Supplementary Movie 2 Movie 1 shown in real time

Supplementary Movie 3 A chain of nickel particles is rotated with a frequency 5 Hz and creates a macroscopic vortex. Bacteria migrate from the origin of rotation due to a shear-induced alignment and self-propulsion. The movie is sped up by a factor of 8.

Supplementary Movie 4 Movie 3 shown in real time

Supplementary Movie 5 A single nickel particle is rotated with a frequency 20 Hz and creates a small depletion zone. Collective behaviour decreases the depletion magnitude created by a single rotating particle.

Supplementary Movie 6 Bacteria were killed by a drop of alcohol. The rotation frequency is 10 Hz. Absence of self-propulsion results in homogeneous spatial distribution of bacteria. The movie is sped up by a factor of 5.

## Figures and Tables

**Table 1:** Number of trapped bacteria versus frequency.

Frequency, Hz	3	20	40
*N*_trap_	4–6	65–70	90–100
Depletion zone radius *R*_d_/*R*	2.2	5.3	6.6
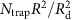	0.82–1.24	2.3–2.5	2–2.3
