# Supplementary material for: Rapid expulsion of microswimmers by a vortical flow
Source: Nat Commun. 2016 Mar 23;7:11114. doi: 10.1038/ncomms11114 (PMC4814579; doi:10.1038/ncomms11114)
Supplement: Supplementary Information — Supplementary Figures 1-6, Supplementary Notes 1-3 and Supplementary References [file ncomms11114-s1.pdf]

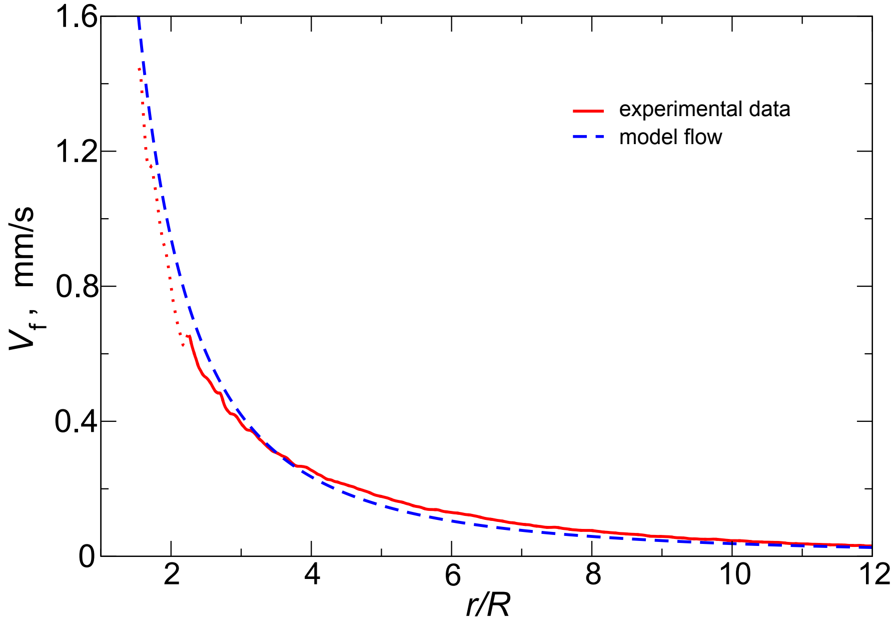

Supplementary Figure 1| Tangential velocity component of the vortical flow. Experimental results are shown in solid red line, rotation frequency is 20 Hz. For small radii $r/R < 2$, tracking of the tracers is affected by rapid flow in the vicinity of the rotating particle. In this region (dashed red line) we have modified the tracking algorithm by implementing prediction of the future positions of the tracers. Dashed blue line displays the theoretical dependence $V_{\mathrm{f}}(r) = \omega R^3/r^2$ which was used in our numerical analysis. Radius of the particle is $R = 30\mu m$. The theoretical curve fits the experimental data reasonably well.

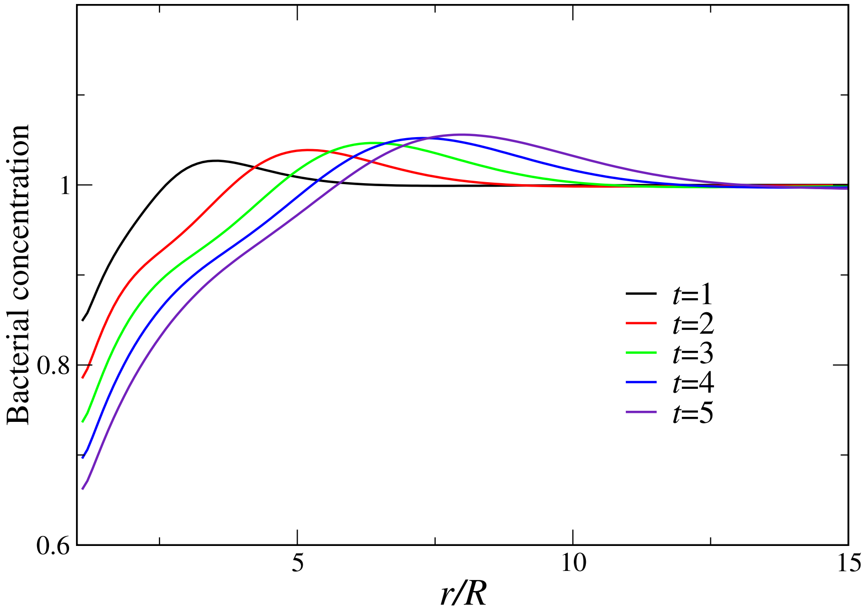

Supplementary Figure 2| Evolution of the bacterial concentration profiles vs time for rotational diffusion $D_{\mathrm{r}} = 0.2$, frequency is $f = 3$Hz and translational diffusion is $D_{\mathrm{t}} = 0.2$. The concentration profiles $\rho(r)$ were obtained by numerical solution of Supplementary Equation (1) for no-flux boundary conditions and initial condition $P(r, \psi, t = 0) = 1$. A propagating peak is formed after the onset of rotation.

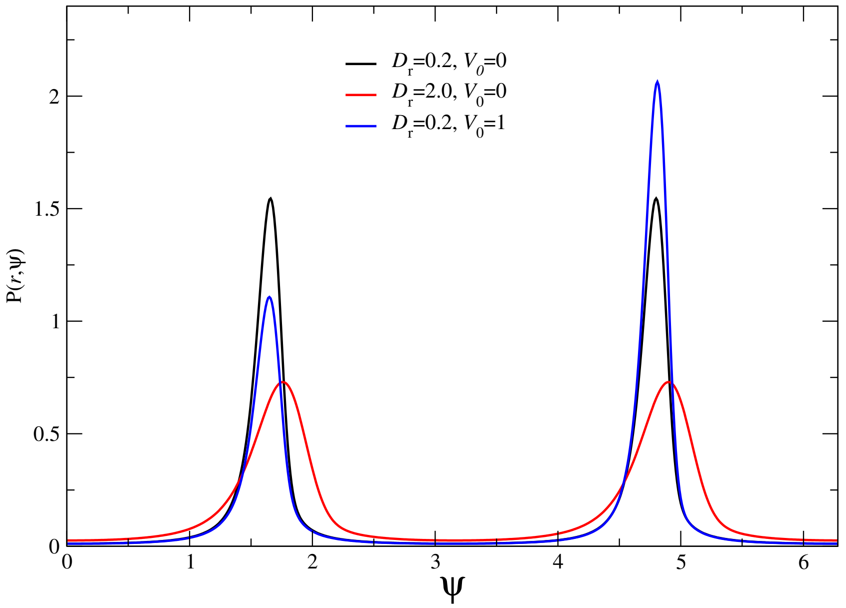

Supplementary Figure 3| Distribution functions $P(r, \psi)$ for $W = \omega R^2/D_\mathrm{r} = 1250$ and $r = 2$ for different values of the dimensionless bacterial speed V_0 and the rotational diffusion D_r.

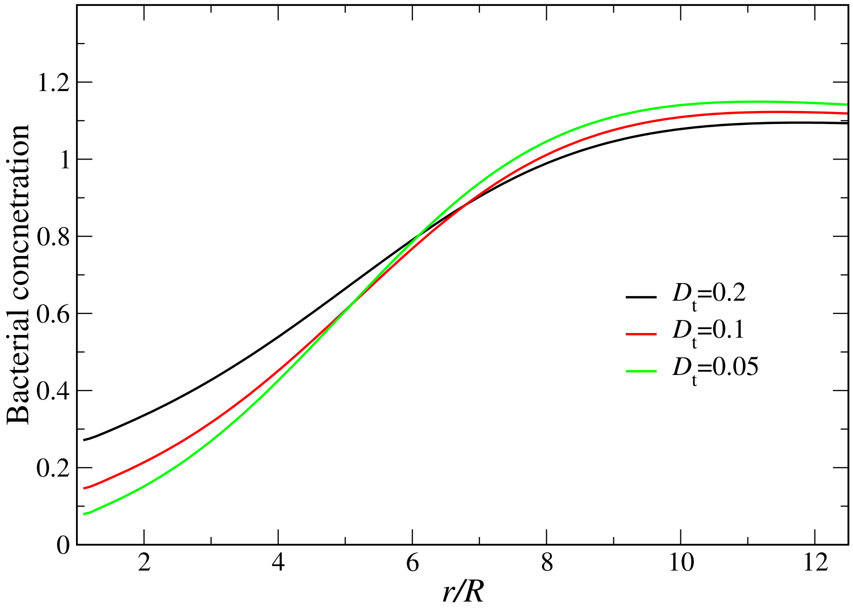

Supplementary Figure 4| Steady-state bacterial concentration profiles for different values of the translational diffusion $D_{\rm t}$. Rotational diffusion is $D_{\rm r} = 0.2$ and frequency is $f = 3$Hz. Decrease in $D_{\rm t}$ results in a slight depression of the concentration ρ in the vicinity of particle.

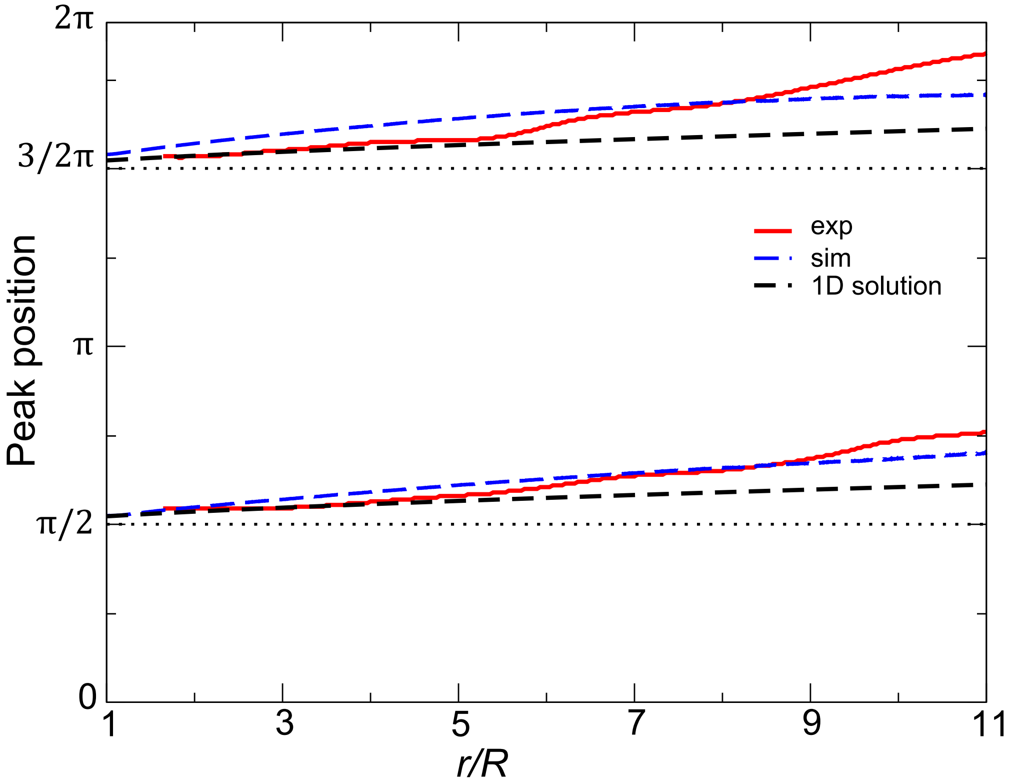

Supplementary Figure 5| Positions of the maxima ψ_m as a function of the distance from the particle. Frequency of rotation is 20 Hz. Red solid lines correspond to experimental data. Blue dashed lines obtained by numerical solution of Supplementary Equation 1. Black dashed lines represent positions of the maxima obtained from Supplementary Equation 4. Rotation diffusion D_{r} value in Supplementary Equations 1, 4 is $D_{\mathrm{r}} = 0.1\ \mathrm{rad}^2\mathrm{sec}^{-1}$.

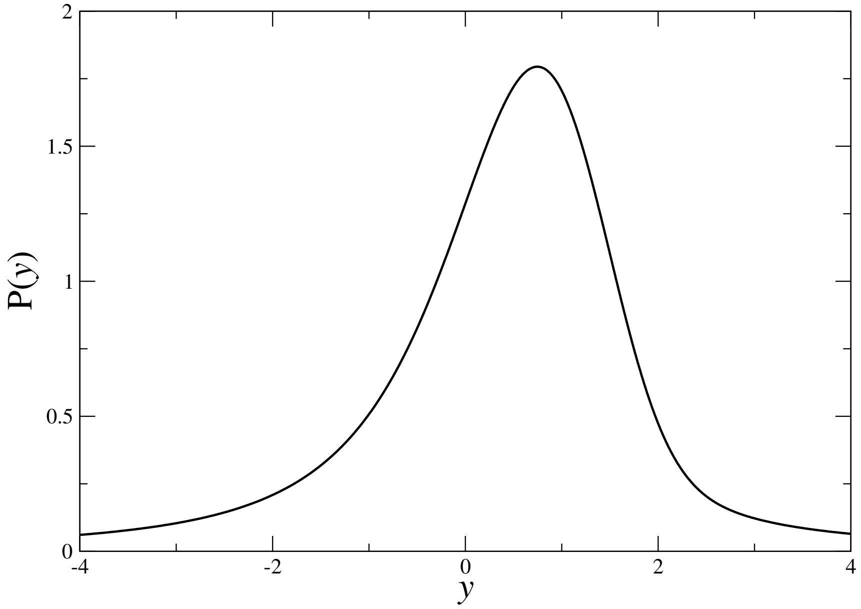

Supplementary Figure 6| Shape function $P(y)$ given by Supplementary Equation (8).

Supplementary Note 1. One-dimensional solution to the Fokker-Plank Equation

The Fokker-Plank equation for the evolution of the probability distribution function $P(r,\psi,t)$ in polar coordinates is of the form

$$\begin{aligned} \partial_t P &= -\frac{1}{r}\partial_r\left(rV_0\cos(\psi)P\right) + D_{\mathrm{t}}\frac{1}{r}\partial_r\left(r\partial_r P\right) \\ &+ \partial_\psi\left(\left(\frac{V_0}{r}\sin(\psi) + \frac{\omega R^2}{r^2}\left(\gamma\cos(2\psi)+1\right)\right)P\right) + D_{\mathrm{r}}\partial_\psi^2 P \end{aligned} \tag{1}$$

Let us consider stationary solution to Supplementary Equation (1). Since for large rotation rates ω the variations in the ψ direction are much faster that in the r direction, we can neglect all terms containing derivatives with respect to r. We call this limit an one-dimensional (1D) approximation. The resulting stationary equation assumes the form

$$\partial_\psi\left(\left(\frac{V_0}{D_{\mathrm{r}}r}\sin(\psi) + \frac{\omega R^2}{D_{\mathrm{r}}r^2}\left(\gamma\cos(2\psi)+1\right)\right)P\right) + \partial_\psi^2 P = 0 \tag{2}$$

In Supplementary Equation (2) the dependence on radius r enters as a parameter. We will verify that the solutions to Supplementary Equation (2) reproduce, on a qualitative level, the angular dependence of the distribution function $P(r,\psi)$. However, the quantitative dependence on the radius r is not necessarily captured correctly due to slow convergence with respect to small parameter $D_{\mathrm{r}}r^2/\omega R^2 \ll 1$.

Supplementary Equation (2) can be integrated once leading to (in the following we set for

simplicity $\gamma = 1$)

$$\left(\left(\frac{V_0}{D_\mathrm{r} r}\sin(\psi) + \frac{\omega R^2}{D_\mathrm{r} r^2}\left(\cos(2\psi)+1\right)\right) P\right) + \partial_\psi P = A \tag{3}$$

where a constant of integration A is determined from 2π periodicity condition of the distribution function. The solution satisfying 2π periodicity condition is of the form

$$P(r,\psi) = \exp(-g(r,\psi))\left(1 - \frac{\int_0^\psi \exp(g(r,\phi))d\phi}{\int_0^{2\pi}\exp(g(r,\phi))d\phi}\left(1-\exp(2\pi W/r^2)\right)\right) \tag{4}$$

where $W = \omega R^2/D_\mathrm{r}$ and

$$g(r,\psi) = \frac{W}{r^2}(\psi + \sin(2\psi)/2) - \frac{V_0}{D_\mathrm{r} r}\cos(\psi) \tag{5}$$

At first sight, Supplementary Equation (4) reproduces angular dependence of the distribution function P reasonably well, see Supplementary Figure 3. In particular, for $V_0 = 0$ the maxima of the distribution function shift right with the increase in the rotational diffusion D_r. As in experiment, for $V_0 > 0$ the maximum near $\psi = 3\pi/2$, corresponding to the stable fixed point of Eq. 5, main text, is higher than that at $\psi = \pi/2$, which corresponds to the unstable point. However, a direct comparison with the numerical solution of Supplementary Equation (1) emphasizes two noticeable differences. First, the width of the peaks obtained from the solution of two-dimensional Supplementary Equation (1) is about twice wider than that given by Supplementary Equation (4). Second, the positions of maxima in Supplementary Eq. (4) are slightly closer to $\psi = \pi/2, 3\pi/2$ (see Supplementary Figure 5) for $r/R > 3.5$. These differences limit the applicability of the 1D approach to a qualitative characterization and small radii r.

Supplementary Note 2. Asymptotic solution for large shear rates

Let us consider asymptotic solutions in the large W limit. In this case the distribution function has two narrow peaks near $\psi = \pi/2, 3\pi/2$. We expand Supplementary Equation (4) near $\psi = \pi/2(3\pi/2) + \xi$, where $\xi \ll 1$. For simplicity, we set $V_0 = 0$. In this case Supplementary Equation (4) becomes

$$Z\xi^2 P + \partial_\xi P = A \tag{6}$$

where $Z = 2W/r^2 = 2\omega R^2/D_\mathrm{r} r^2$. Making a substitution $y = Z^{1/3}\xi$, Supplementary Equation (6) assumes dimensionless form

$$y^2 P + \partial_y P = A \tag{7}$$

Localized solution (shape function) to Supplementary Equation (7) can be written as

$$P = \exp(-y^3/3)\int_{-\infty}^{y}\exp(x^3/3)dx \tag{8}$$

This solution is shown in Supplementary Figure 6. Remarkably, one sees that the maxim is shifted from the origin by $\Delta y \approx 0.74$. Returning to the original scaling, we obtain that in the large shear rate limit $Z \gg 1$, the positions ψ_m of the maxima are given by the following simple expression (for $V_0 = 0$)

$$\psi_m \approx \frac{\pi}{2}\left(\frac{3\pi}{2}\right) + 0.74 Z^{-1/3} = \frac{\pi}{2}\left(\frac{3\pi}{2}\right) + 0.59(D_\mathrm{r} r^2/\omega R^2)^{1/3} \tag{9}$$

By fitting the experimental data with Supplementary Equation (9), we can estimate the diffusion coefficient D_r. The fit for small radii ($r/R < 3.5$) yields a realistic value of the diffusion $D_\mathrm{r} \approx 0.1$ rad^2/sec. For larger r values the fit becomes inaccurate, and gives an order of magnitude large value of D_r.

Supplementary Note 3. Estimation of the effective viscosity reduction

The relative effective viscosity η_eff of a suspension of microswimmers in the dilute limit can be written as following

$$\frac{\eta_\mathrm{eff}}{\eta} = 1 + \frac{\sigma_\mathrm{shear}^\mathrm{active}}{\dot\gamma} \tag{10}$$

where η is the solvent dynamic viscosity, $\sigma_\mathrm{shear}^\mathrm{active}$ is the shear component of the stress tensor associated with swimming particles and normalized by the solvent viscosity η, $\dot\gamma$ is the shear strain rate. Here we neglect the stress contributions from passive inclusions and from fluctuations, see Ref.[1]. This approximation is valid in the limit of vanishing rotational diffusion and small concentration of swimmers.

According to Ref.[1], the active stress contribution in two dimensions can be written as

$$\sigma_{ij}^\mathrm{active}(x,y) = -a_0 \int (\delta_{ij} - 2 d_i d_j)\, P(x,y,\theta) d\theta \tag{11}$$

Here a_0 is the strength of the hydrodynamic dipole (stresslet) imposed by the bacteria on the fluid, and d_i is the component of the unit vector of bacterial orientation, $d_x = \cos\theta, d_y = \sin\theta$. For bacteria (pusher-like swimmers) $a_0 < 0$. Note that here the dipole strength is normalized by the solvent viscosity η.

To estimate the viscosity reduction for a rotational flow, we need to calculate the $r\varphi$ component of the stress tensor σ^active in polar coordinate system. We convert the stress tensor from Cartesian to polar basis according to the following relation

$$\begin{bmatrix} \sigma_{rr} & \sigma_{r\varphi} \\ \sigma_{\varphi r} & \sigma_{\varphi\varphi} \end{bmatrix} = \begin{bmatrix} \cos\varphi & \sin\varphi \\ -\sin\varphi & \cos\varphi \end{bmatrix} \begin{bmatrix} \sigma_{xx} & \sigma_{xy} \\ \sigma_{yx} & \sigma_{yy} \end{bmatrix} \begin{bmatrix} \cos\varphi & -\sin\varphi \\ \sin\varphi & \cos\varphi \end{bmatrix}$$

Taking into account that $\sigma_{xy} = \sigma_{yx}, \sigma_{r\varphi} = \sigma_{\varphi r}$, the component $\sigma_{r\varphi}^\mathrm{active}$ assumes the form

$$\sigma_{r\varphi}^\mathrm{active} = a_0 \int_0^{2\pi} \sin(2\psi) P(r,\psi) d\psi \tag{12}$$

where $\psi = \theta - \phi$ is the relative angle. In the limit of small fluctuations, $D_\mathrm{r} \ll |\dot\gamma|$, the steady-state probability distribution $P(r,\psi)$ is represented by two very narrow peaks of different height at the angles $\psi \approx \psi_{1,2}$, see Supplementary Figure 3. Thus, in the small diffusion limit, the steady-state probability distribution function $P(r,\psi)$ can be replaced by a δ-function, $P(r,\psi) \approx n(r)\delta(\psi - \psi_1(r))$, where ψ_1 is the stable fixed point given by $\psi_1 \approx 3\pi/2 + \sqrt{V_0 r/2\omega R^2}$, and n is the local concentration of bacteria. Substituting this expression for $P(r,\psi)$ into Supplementary Equation (12), we readily obtain

$$\sigma_{r\varphi}^\mathrm{active} \sim a_0 n \sin(2\psi_1) \approx |a_0| n \sqrt{2V_0 r/\omega R^2} > 0 \tag{13}$$

For the flow around a rotating particle $V_\varphi = \omega R^2/r$, the corresponding shear strain rate is $\dot\gamma = -\omega R^2/r^2 < 0$. Since for this direction of the flow the active stress contribution is positive and the corresponding strain rate is negative, we obtain the overall reduction of the effective viscosity:

$$\frac{\eta_\mathrm{eff}(r)}{\eta} = 1 - |a_0| n \frac{\sqrt{2V_0/r}}{|\dot\gamma|^{3/2}} \tag{14}$$

Note a non-Newtonian rheology of the active suspension described by Supplementary Equation (14): the effective viscosity depends on the strain rate. For very small strain rates, $\dot{\gamma} \to 0$, the effective viscosity $\eta_{\mathrm{eff}} \to -\infty$. However, this limit is singular, and an infinitesimally small rotational diffusion D_{r} will regularize the divergence, see Ref. [1].

Now we estimated the effective viscosity for the conditions of the experiment in Ref.[2], i.e for the concentration $n \leq 10^{10}$ cm^{-3} (i.e. slightly below the threshold for the onset of collective behavior), and for the rotation rate of 0.5 Hz, particle radius $R \approx 30$ μm, and swimming speed $V_0 \approx 30$ μm s^{-1}. The magnitude of the dipole moment a_0 can be extracted from Ref. [3], that reported for E.coli bacteria the value of $|a_0| \approx 32$ μm^{3}s^{-1}. For Bacillus subtilis we expect a_0 to be at least twice higher due to the increased size and the swimming speed, i.e. $|a_0| \approx 60$ μm^3 s^{-1}. Near the particle, i.e. for $r \approx R$,and for the above experimental values we obtain from Supplementary Equation (14) the following estimate for the relative effective viscosity: $\eta_{\mathrm{eff}}/\eta \approx 0.7 - 0.8$. Thus, relative reduction of the effective viscosity due to this effect is of the order 20-30%. These values are somewhat below the corresponding experimental values. Further reduction of the effective viscosity is likely due to additive effects of fluctuations and hydrodynamic interactions between the bacteria, see Refs. [1,4]. Thus, in the case of flow around a rotating particle, the vortical structure of the flow results in strong asymmetry of the orientation distribution function, that, in turn, contributes to the viscosity reduction.

Supplementary References

1. Haines, B. M., Sokolov, A., Aranson, I. S., Berlyand, L. & Karpeev, D. A. Three-dimensional model for the effective viscosity of bacterial suspensions. *Phys. Rev. E* **80**, 041922 (2009).

2. Sokolov, A. & Aranson, I. S. Reduction of viscosity in suspension of swimming bacteria. *Phys. Rev. Lett.* **103**, 148101 (2009).

3. Drescher, K., Dunkel, J., Cisneros, L. H., Ganguly, S. & Goldstein, R. E. Fluid dynamics and noise in bacterial cell–cell and cell–surface scattering. *Proc. Natl. Acad. Sci.* **108**, 10940–10945 (2011).

4. Ryan, S. D., Haines, B. M., Berlyand, L., Ziebert, F. & Aranson, I. S. Viscosity of bacterial suspensions: Hydrodynamic interactions and self-induced noise. *Phys. Rev. E* **83**, 050904 (2011).
